# Unveiling the molecular landscape of PCOS: identifying hub genes and causal relationships through bioinformatics and Mendelian randomization

**DOI:** 10.3389/fendo.2024.1431200

**Published:** 2024-12-13

**Authors:** Yifang He, Yanli Wang, Xiali Wang, Shuangping Deng, Dandan Wang, Qingqing Huang, Guorong Lyu

**Affiliations:** ^1^ Department of Ultrasound, The Second Affiliated Hospital of Fujian Medical University, Quanzhou, China; ^2^ Departments of Medical Imaging, Quanzhou Medical College, Quanzhou, China

**Keywords:** pcos, bioinformatic analysis, Mendelian randomization, causal relationship, SNP

## Abstract

**Background:**

Polycystic ovary syndrome (PCOS) is a complex endocrine disorder with various contributing factors. Understanding the molecular mechanisms underlying PCOS is essential for developing effective treatments. This study aimed to identify hub genes and investigate potential molecular mechanisms associated with PCOS through a combination of bioinformatics analysis and Mendelian randomization (MR).

**Methods:**

This study employed bioinformatics analysis in conjunction with MR methods using publicly available databases to identify hub genes. We employed complementary MR methods, including inverse-variance weighted (IVW), to determine the causal relationship between the hub genes and PCOS. Sensitivity analyses were performed to ensure results reliability. Enrichment analysis and immune infiltration analysis were further conducted to assess the role and mechanisms of hub genes in the development of PCOS. Additionally, we validated hub gene expression in both an animal model and serum samples from PCOS patients using qRT-PCR.

**Results:**

IVW analysis revealed significant associations between 10 hub genes and the risk of PCOS: CD93 [*P*= 0.004; *OR* 95%*CI*= 1.150 (1.046, 1.264)], CYBB [*P*= 0.013; *OR* 95%*CI*= 1.650 (1.113,2.447)], DOCK8 [*P*= 0.048; *OR* 95%*CI*= 1.223 (1.002,1.494)], IRF1 [*P*= 0.036; *OR* 95%*CI*= 1.343 (1.020,1.769)], MBOAT1 [*P*= 0.033; *OR* 95%*CI*= 1.140 (1.011,1.285)], MYO1F [*P*= 0.012; *OR* 95%*CI*= 1.325 (1.065,1.649)], NLRP1 [*P*= 0.020; *OR* 95%*CI*= 1.143 (1.021,1.280)], NOD2 [*P*= 0.002; *OR* 95%*CI*= 1.139 (1.049,1.237)], PIK3R1 [*P*= 0.040; OR 95%*CI*= 1.241 (1.010,1.526)], PTER [*P*= 0.015; *OR* 95%*CI*= 0.923 (0.866,0.984)]. No heterogeneity and pleiotropy were observed. Hub genes mainly enriched in positive regulation of cytokine production and TNF signaling pathway, and exhibited positive or negative correlations with different immune cells in individuals with PCOS. qRT-PCR validation in both the rat model and patient serum samples confirmed hub gene expression trends consistent with our combined analysis results.

**Conclusions:**

Our bioinformatics combined with MR analysis revealed that CD93, CYBB, DOCK8, IRF1, MBOAT1, MYO1F, NLRP1, NOD2, PIK3R1 increase the risk of PCOS, while PTER decreases the risk of PCOS. This discovery has implications for clinical decision-making in terms of disease diagnosis, prognosis, treatment strategies, and opens up novel avenues for drug development.

## Introduction

Polycystic ovary syndrome (PCOS) is the most common endocrine disorder in women of reproductive age, with a global prevalence estimated at 5-20%. This multifaceted condition affects reproductive, metabolic, and psychological aspects throughout an individual’s lifespan (1, 2). Women with PCOS commonly experience irregular menstrual cycles, elevated levels of male hormones (hyperandrogenism), enlarged ovaries with small cysts, and metabolic disturbances such as insulin resistance and obesity (3). Beyond reproductive health, PCOS increases the risk of various health challenges, including type 2 diabetes, cardiovascular disease, infertility, and immune response dysregulation (4–6).

Despite extensive research over several decades, the precise causes and mechanisms behind the development and progression of PCOS remain enigmatic. This disorder is widely acknowledged to have a multifactorial etiology, involving intricate interplay among genetic, hormonal, and environmental factors (7–9). Genetic studies have identified several susceptibility loci linked to PCOS, suggesting a genetic predisposition to the disorder (10). However, the specific genes and molecular pathways driving PCOS remain incompletely understood.

Recent advances in high-throughput technologies and the abundance of large-scale genomic data have opened up new avenues to uncover the complex molecular landscape of PCOS (11). The utilization of bioinformatics analysis, in conjunction with computational and statistical methods, has emerged as a valuable approach to explore and interpret these extensive datasets. By integrating diverse data sources and employing advanced algorithms, bioinformatics analysis enables the identification of differentially expressed genes (DEGs), facilitates functional enrichment analysis, and facilitates the construction of protein-protein interaction (PPI) networks. These techniques contribute to the discovery of pivotal genes and pathways associated with PCOS (12).

Distinguishing between correlation and causation poses a significant challenge when investigating the causal relationship between genes and diseases. Mendelian randomization (MR) analysis has e become a powerful tool for inferring causal associations by utilizing single nucleotide polymorphisms (SNPs) as instrumental variables (IVs) (13, 14). By exploiting SNPs strongly associated with a particular exposure, MR analysis can provide valuable evidence of causality, thereby shedding light on the intricate interplay among genes, environmental factors, and disease phenotypes (15). The application of MR analysis in PCOS research holds the potential to unravel the causal effects of key genes or pathways on the development and progression of PCOS.

To our knowledge, no previous study has combined bioinformatics and MR analysis to identify hub genes and elucidate potential molecular mechanisms in PCOS. By integrating gene expression datasets and using genetic variation as instrumental variables (IVs) in MR analysis, this approach aims to identify PCOS-associated genes with a causal relationship. Through functional enrichment analysis and the use of tools like CIBERSORT, this approach enables us to gain insights into the biological functions, pathways, and immune cell infiltration signatures associated with these hub genes, providing a more comprehensive understanding of the molecular landscape of PCOS.

This study’s findings will enhance our understanding of the underlying molecular mechanisms implicated in PCOS and their relevance to developing innovative therapeutic strategies. By identifying hub genes and investigating causal relationships, this study sheds light on the complex origins of PCOS and highlights new therapeutic targets. Ultimately, this research may pave the way for personalized approaches to managing PCOS, improving clinical outcomes and enhancing the quality of life for affected individuals.

## Materials and methods

### Bioinformatics analysis

#### Data collection and preprocessing

Four GEO datasets (GSE102293, GSE137684, GSE34526, and GSE5850) containing (granulosa cell) GC samples of 23 PCOS patients and 17control subjects were merged into a single unified dataset. To minimize the impact of batch effects, we applied the ComBat function from the sva R package (16), specifying known batch variables, including dataset sources and technical variations. This allowed the ComBat algorithm to adjust expression values based on these predefined batch differences, effectively harmonizing the data and removing batch-specific biases.

#### Identification of DEGs

We identified DEGs between PCOS patients and controls using the *limma* R package, with criteria of *P* < 0.05 and a minimum log fold change of 0.585. The *ggplot2* R package was utilized to create a volcano plot, while a heatmap represented the top 50 up-regulated and down-regulated DEGs.

### Circos plot and enrichment analysis

To visualize the genomic distribution the hub genes, we generated circos plots with the *circlize* R packages. Functional analyses were conducted using the *clusterProfiler* R package for Gene Ontology (GO) and Kyoto Encyclopedia of Genes and Genomes (KEGG) pathway. Significant enrichment in biological functions and pathways were determined by KEGG with a P-value < 0.05 and GO with a Q-value < 0.05 to account for multiple testing.

### Immune infiltration estimations via CIBERSORT

This analysis evaluated the correlation between the expression of hub genes and immune-related functions. We categorized hub genes into high and low groups based on cutoff values. Immune cell infiltrations were quantified using the *gsva* package, and correlation with immune infiltrating cells and functions were assessed in the GEO cohort (17). Additionally, CIBERSORT, an algorithm based on gene expression, was employed to evaluate immune cell infiltration signatures, and analyze immune infiltration patterns in PCOS samples (18). We used CIBERSORT to determine immune cell type proportions in PCOS samples, exploring correlations between hub genes and immune cell populations. Spearman correlation coefficients calculated with the R statistical package, and results were visualized using the *ggplot2* package in R.

### Mendelian randomization study

#### Genome-wide association study data sources

The GWAS summary data for gene traits can be accessed from the GWAS catalog (https://gwas.mrcieu.ac.uk) with accession numbers ranging from ENSG00000000003 to ENSG00000270184 (**Supplementary Table S1** **).** The expression quantitative trait locus (eQTL) genes expression, including 11 million SNPs, was obtained from 31,684 European blood samples across 37 eQTLGen consortium cohorts (19). Furthermore, the specific GWAS statistics data for PCOS, released by Tyrmi JS et al. in January 2022, was accessed from the project website (https://gwas.mrcieu.ac.uk/datasets/ebi-a-GCST90044902/) (20). The diagnostic criteria for PCOS followed ICD-10, ICD-9, or ICD-8 standards, requiring at least two out of the following: chronic anovulation, hyperandrogenism, or ultrasound-confirmed polycystic ovaries (21). This dataset includes genetic variant information from 797 PCOS cases and 140,558 controls, comprising a total of 22,981,890 genetic variants.

#### Selection of instrumental variables

IVs play a fundamental role in MR analysis, enabling the estimation of causal relationships between exposures and outcomes. Three key criteria were used to establish valid IVs:1. The IVs should exhibit a strong correlation with the exposure factors of interest; 2. The IVs must not be associated with any confounding factors that could potentially influence the outcome; 3.The IVs should solely affect the results through the exposure factors, without any direct influence on the outcome.

IVs are selected through the following steps (1): Identifying SNPs exhibit a strong association with the exposure factor, using a significance threshold of *P* < 5×10^-8^; (2) SNPs in linkage disequilibrium (LD) are excluded based on the criteria of r^2^ = 0.001 and kb = 10,000; (3) The F-statistic (β^2^/SE^2^) is calculated to assess weak IV bias. F-values below 10 indicate weak IV bias, which can potentially result in an underestimation of statistical power (22). Besides, we utilized the online tool PhenoScanner (http://www.phenoscanner.medschl.cam.ac.uk/) to search and assess all known relevant phenotypes considered in our analysis. This step helps ensure that the chosen IVs are specific to the exposure of interest and minimize the potential for pleiotropy. IVs were initially selected using the *TwoSampleMR* software package.

#### Date analysis

We conducted MR analysis to explore the causal relationships between the exposure genes and PCOS using the *TwoSampleMR* R package. Multiple MR methods were applied to estimate causal effects: MR Egger (23), weighted median (24), inverse variance-weighted (IVW) (13), simple mode, and weighted mode, with IVW as the primary method due to its robustness under certain assumptions (25). Sensitivity analysis was performed by excluding one SNP at a time utilizing the leave-one-out method to validate the effectiveness and robustness of the IVW results. Heterogeneity were assessed with MR Egger and IVW methods, using Cochrane’s Q test, where a p-value greater than 0.05 suggests a lack of significant diversity. Horizontal pleiotropy, where IVs might affect the outcome through pathways unrelated to the exposure, was assessed using the MR-Egger intercept test, and a p-value greater than 0.05 indicates no evidence of horizontal pleiotropy (26). Deviation from the null intercept suggests the presence of pleiotropic effects. Funnel plots and forest plots were generated to visually assess precision, effect sizes, and causal estimates.

#### Selection of hub genes

Firstly, we filtered PCOS-related genes by the condition of IVW p-value < 0.05 and pleiotropy test p-value > 0.05. These genes were further analyzed in conjunction with the DEGs obtained from the bioinformatics analysis. The intersection of upregulated DEGs with an odds ratio (OR) > 1 in MR analysis and downregulated DEGs with an OR < 1 in MR analysis was obtained to identify hub genes, which may play essential roles in the pathogenesis of PCOS and serve as potential therapeutic targets or diagnostic markers.

### Construction of PCOS rat model, clinical sample collection, and experimental validation

#### Animals

The animal care and experiments complied with the guidelines of the Chinese Animal Experimentation Committee and were approved by the Laboratory Animal Institutional Review Committee of Quanzhou Medical College (No. 2024017), ensuring the ethical treatment of animals throughout the study. A total of 24 female Sprague-Dawley rats at 3 weeks of age (SD, 55 ± 3g) were acquired from Fuzhou Houji Experimental Animal Center. All animals were housed in specific pathogen-free (SPF) grade plastic cages with a 12-hour light/dark cycle, temperature ranging from 20-24°C, humidity at 50%, and provided with unrestricted access to sterilized food and water. Bedding for these animals was replaced twice a week.

### Construction of PCOS rat model and sample collection

The rats were randomly allocated into PCOS group (n=12) and control group (n=12) using a random number generator to ensure unbiased distribution. The PCOS model was established using DHEA (Shanghai Yuanye Biotechnology Co., LTD, Shanghai, China), which was chosen based on previous studies demonstrating its efficacy in inducing PCOS-like symptoms in rats (27). DHEA (6mg/100g) was dissolved into 0.2ml sesame oil (Yuanye, Shanghai, China) according to rat body weight and injected subcutaneous into the neck and back for 23 days. The control group received 0.2ml sesame oil via the same route and duration.

Six randomly selected rats from each group had their vaginal secretions collected by cell smearing during the last 7 days of the modeling period at 9:00 AM. After the modeling period, all rats were anesthetized with sodium pentobarbital (Nembutal, Ovation Pharmaceuticals Inc. Deerfield, USA) via intraperitoneal injection. Blood samples were obtained via cardiac puncture, followed by centrifugation at 4°C for 20 minutes to collect the serum. The obtained serum was subsequently stored at -80°C for further analysis. Ovarian tissues were collected, with one side immersed in 4% paraformaldehyde and the other side stored in liquid nitrogen. Finally, animals were euthanized with sodium pentobarbital, ensuring humane treatment throughout the study.

### Clinical serum sample collection

In addition to the animal model, serum samples were collected from 8 patients diagnosed with PCOS and 8 healthy controls, recruited from the Second Affiliated Hospital of Fujian Medical University. This choice of human serum samples aligns with the sample source used in the MR analysis. The study was approved by the Institutional Review Board of the Second Affiliated Hospital of Fujian Medical University (No. 2023357). All participating women provided written informed consent. The study included women diagnosed with PCOS based on the Rotterdam Criteria (21), consistent with the diagnostic criteria used in the GEO database and MR analysis. The control group consisted of women with regular menstrual cycles, no clinical signs of hyperandrogenism, and normal ovarian morphology on ultrasonography. Both the PCOS group and control group included women aged ≤42 years and excluded individuals with abnormal thyroid function, type 2 diabetes, Cushing’s syndrome, congenital adrenal hyperplasia, hyperprolactinemia, premature ovarian failure, recent medication use (e.g., birth control pills and glucocorticoids), ovarian tumors, or unwillingness to participate. Blood samples from these individuals were collected and centrifuged at 4°C for 20 minutes to obtain the serum, then stored at -80°C for subsequent qRT-PCR analysis.

### Ovarian hematoxylin and eosin staining, vaginal smears, and serum analysis

After fixation, rat ovarian specimens were embedded in paraffin, and 4 μm sections were prepared for HE staining using a kit from Beijing Solarbio Science and Technology Co., Ltd (Beijing, China) to observe pathological changes in ovarian structure and morphology. To assess the estrous cycle phases, proestrus (P), estrus (E), metestrus (M), and diestrus (D), vaginal smears were obtained by applying vaginal secretions onto glass slides and stained with the same HE kit. Serum samples from the rats were collected to measure sex hormone levels, including progesterone (PRGE), prolactin (PRL), estradiol (E2), follicle-stimulating hormone (FSH), luteinizing hormone (LH), testosterone (T), and anti-Mullerian hormone (AMH), using an ELISA kit from MeiMian Biotech Co., Ltd (Jiangsu, China), following the manufacturer’s instructions.

### Quantitative real-time PCR analysis

The mRNA expression levels of Cd93, Cybb, Dock8, Irf1, Mboat1, Myo1f, Nlrp1, Nod2, Pik3r1, and Pter were analyzed using qRT-PCR. Total RNA was purified using the RNA-quick Purification Kit (ESscience Biotech Co., Ltd, Shanghai, China), and the concentration and purity of the RNA were determined using a nucleic acid protein detector (Applied Biosystems, Carlsbad, USA). Subsequently, reverse transcription was performed using the PrimeScript™ RT Reagent Kit with gDNA Eraser (TaKaRa Bio Inc., Tokyo, Japan). qRT-PCR was conducted using the TB Green^®^ Premix Ex Taq™ (Tli RNaseH Plus) and 10 μM primer (final concentration) with SYBR, following the manufacturer’s instructions (TaKaRa). The primer sequences for the target genes were synthesized by Sangon Biotechnology Co., Ltd. (**Supplementary Table S2**). For both the rat and clinical human samples, the expression of the target genes was normalized to the GAPDH gene using the 2-ΔΔCT-method. Each reaction was conducted in triplicate.

### Statistical analysis

GraphPad Prism 9.0 (GraphPad software Inc., San Diego, CA, USA) evaluates statistical data. The two groups were compared using the Student’s t-test, and a *P* value less than 0.05 was considered statistically significant.

## Results

### Acquisition of DEGs related to PCOS

**Figure 1:**
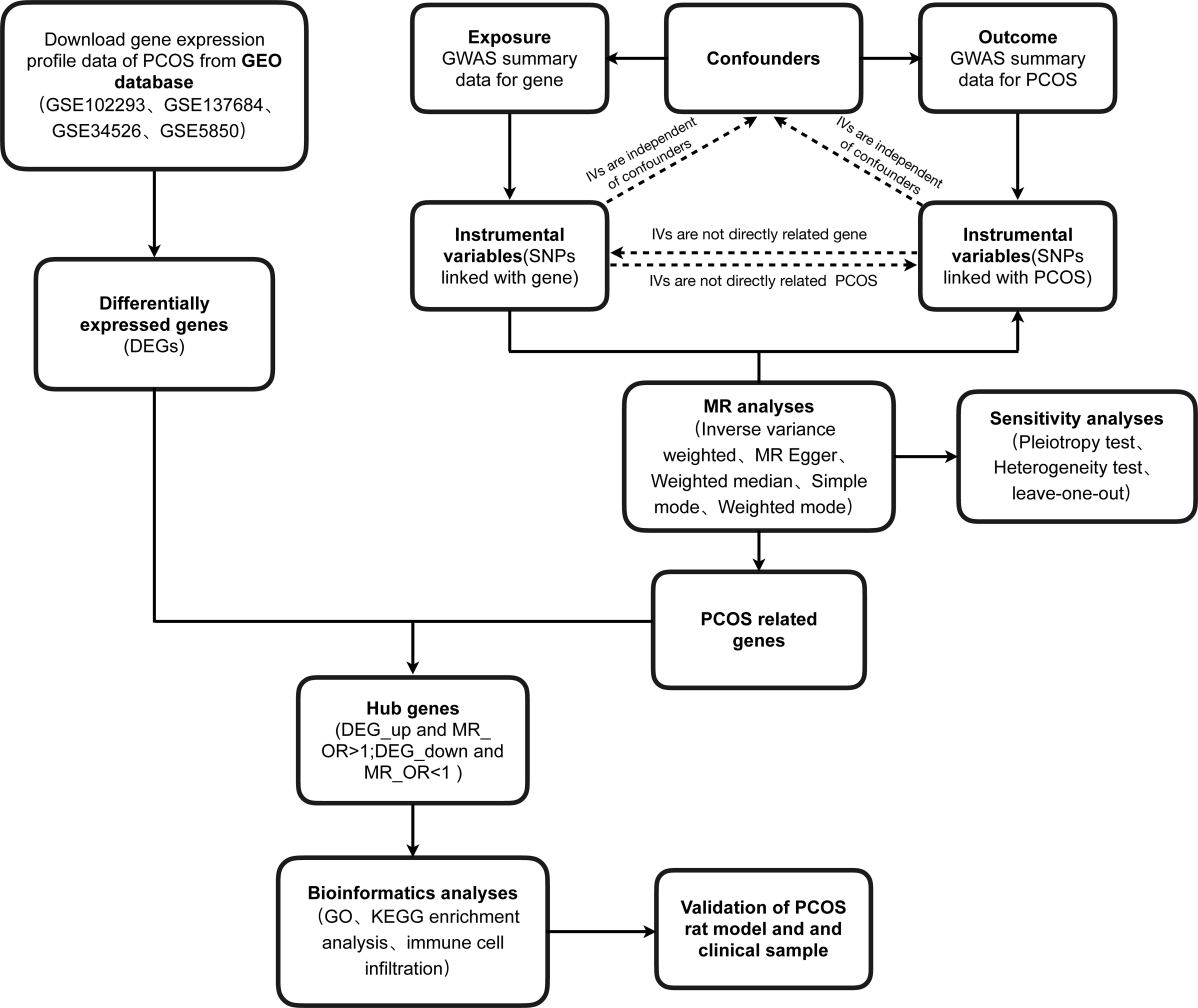
Workflow chart of data generation and analysis.

**Figure 2:**
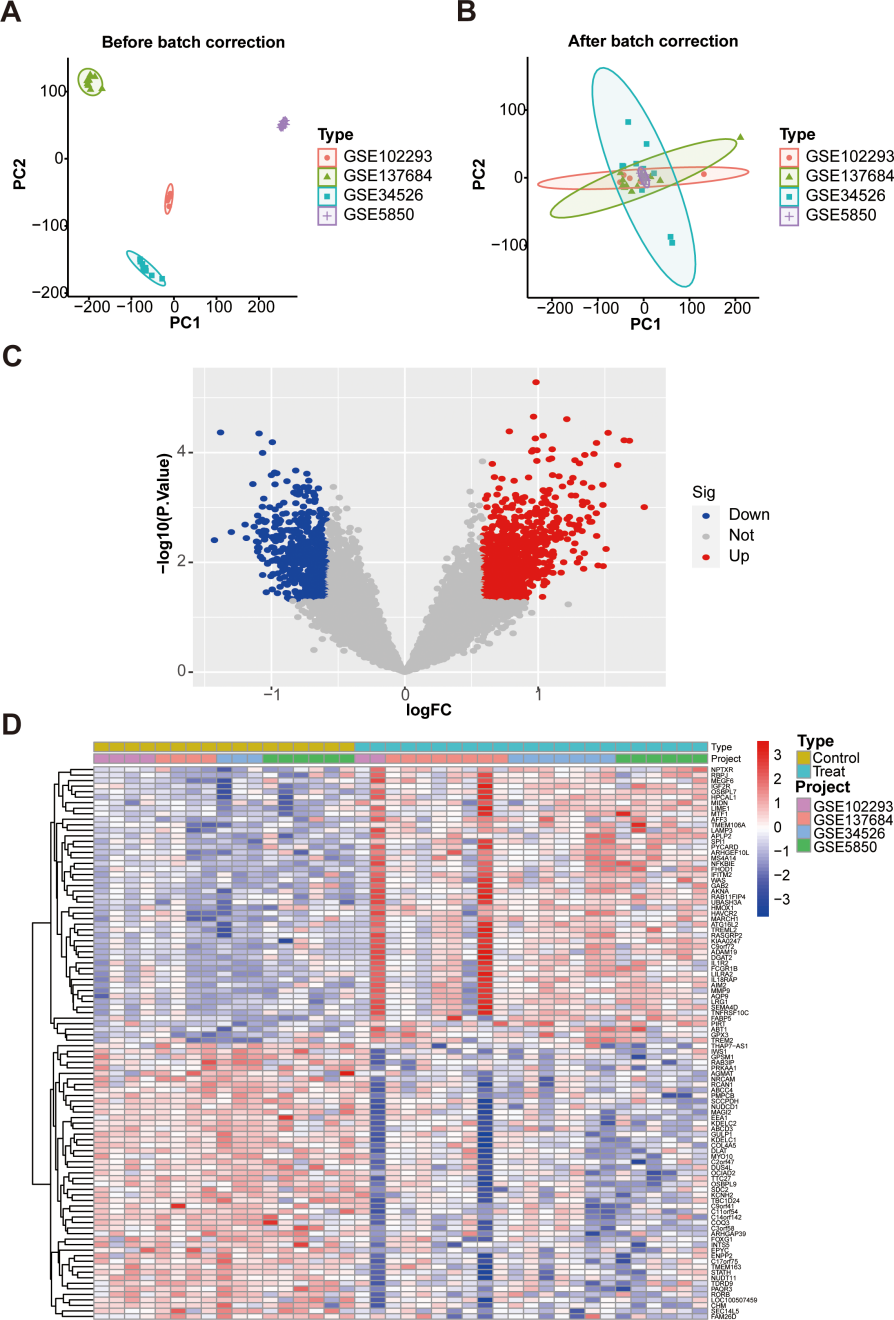
Identification of DEGs in GCs associated with PCOS patients and control subjects. **(A)** PCA plot of GC samples before batch correction; **(B)** PCA plot after batch correction; **(C)** The volcano plot. Red represent up-regulated genes, gray dots represent not significant genes, blue dots represent down-regulated genes; **(D)** Heat map. DEGs, differentially expressed genes. PCA, principal component analysis.

Firstly, the general workflow of this study is depicted in **Figure 1**. Our analysis utilized an integrated dataset comprising the results from multiple studies, including GSE102293, GSE137684, GSE34526, and GSES850. By applying our batch correction approach, we consolidated these datasets, observing a significant reduction in inter-batch variability. This adjustment enabled a comprehensive investigation of gene expression differences between individuals with PCOS and healthy control GCs (**Figures 2A, B**; **Supplementary Table S3**). Through screening criteria, namely *P* value < 0.05 and |logFC| > 0.585, we identified total of 1428 DEGs. More detailed information for these DEGs see **Supplementary Table S4**. Volcano plot (**Figure 2C**) and heatmap (**Figure 2D**) visually depict the distinct expression patterns of these DEGs.

### Causal association between exposure genes and PCOS

From the 19,942 GWAS summary data, SNPs that exhibited linkage disequilibrium were removed from the original GWAS data. Following our predefined criteria, we selected specific SNPs as IVs for their corresponding exposure genes. To enhance the reliability of our findings, we excluded weak IVs, and all SNPs included in our study demonstrated F statistics greater than 10 (**Supplementary Tables S5**, **S6**). By Searching PhenoScanner, we confirmed that there were no obesity-associated SNPs in IVs. This further strengthens the validity of our IVs selection and reduces the potential for confounding effects related to obesity in our analysis.

Subsequently, we conducted MR analysis, and further performed heterogeneity and pleiotropy tests (**Supplementary Tables S7**-**S9**). PCOS-related genes were obtained through the criteria of *P* value < 0.05 for IVW and *P* value > 0.05 for pleiotropy tests (**Supplementary Table S10**).

### Acquisition of hub genes related to PCOS and MR results

**Figure 3:**
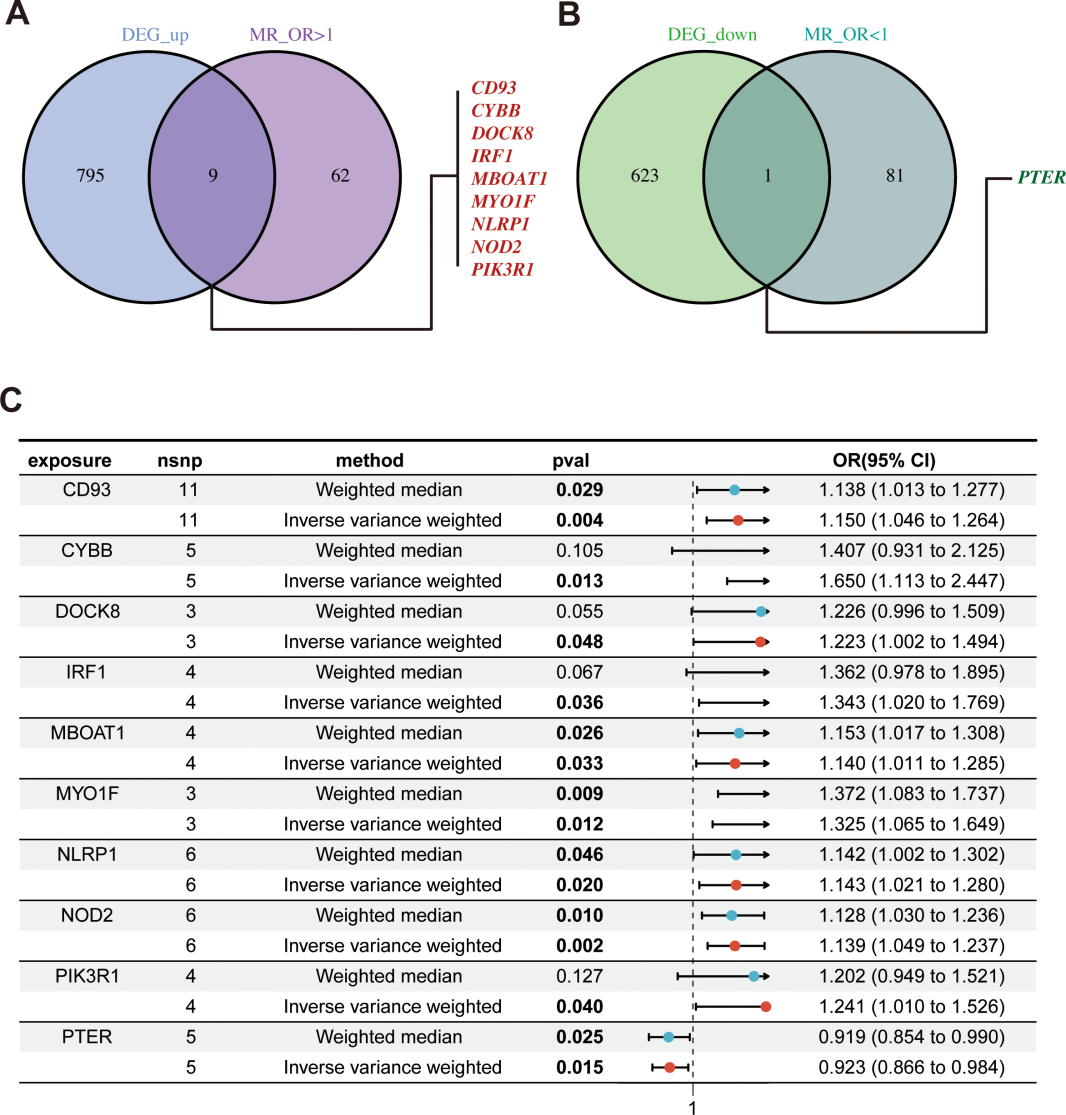
Identify of hub genes and MR results; **(A)** Venn diagram showed up-regulated hub genes; **(B)** Venn diagram showed down-regulated hub genes; **(C)** The forest plot of MR results for 10 hub genes and PCOS. MR, mendelian randomization.

**Figure 4:**
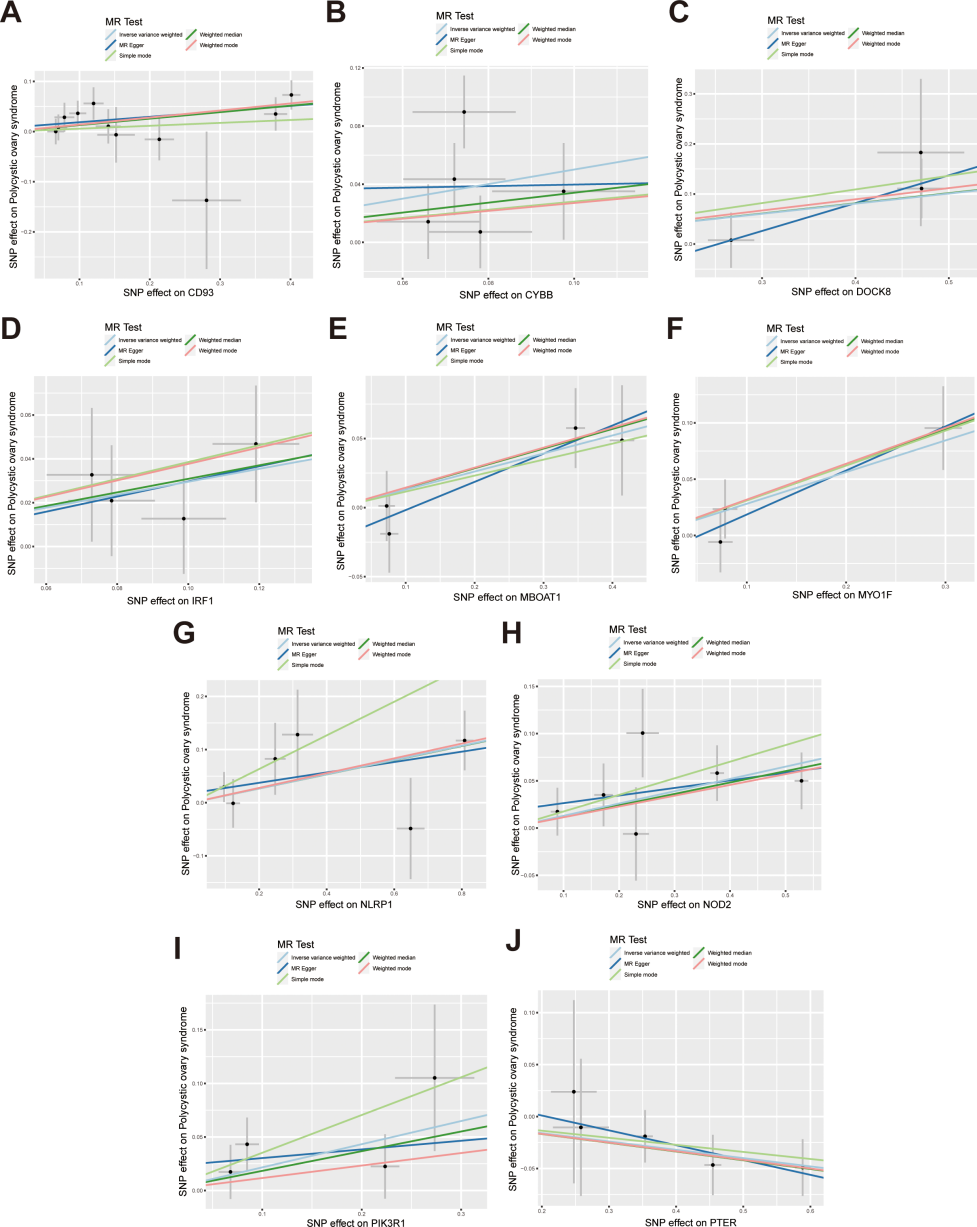
Scatter plots illustrate the causal effects of hub genes on PCOS. **(A-I)** Scatter plots showed that CD93, CYBB, DOCK8, IRF1, MBOAT1, MYO1F, NLRP1, NOD2, PIK3R1 significantly increased the risk of PCOS; **(J)** Scatter plots showed that PTER significantly reduced the risk of PCOS.

**Figure 5:**
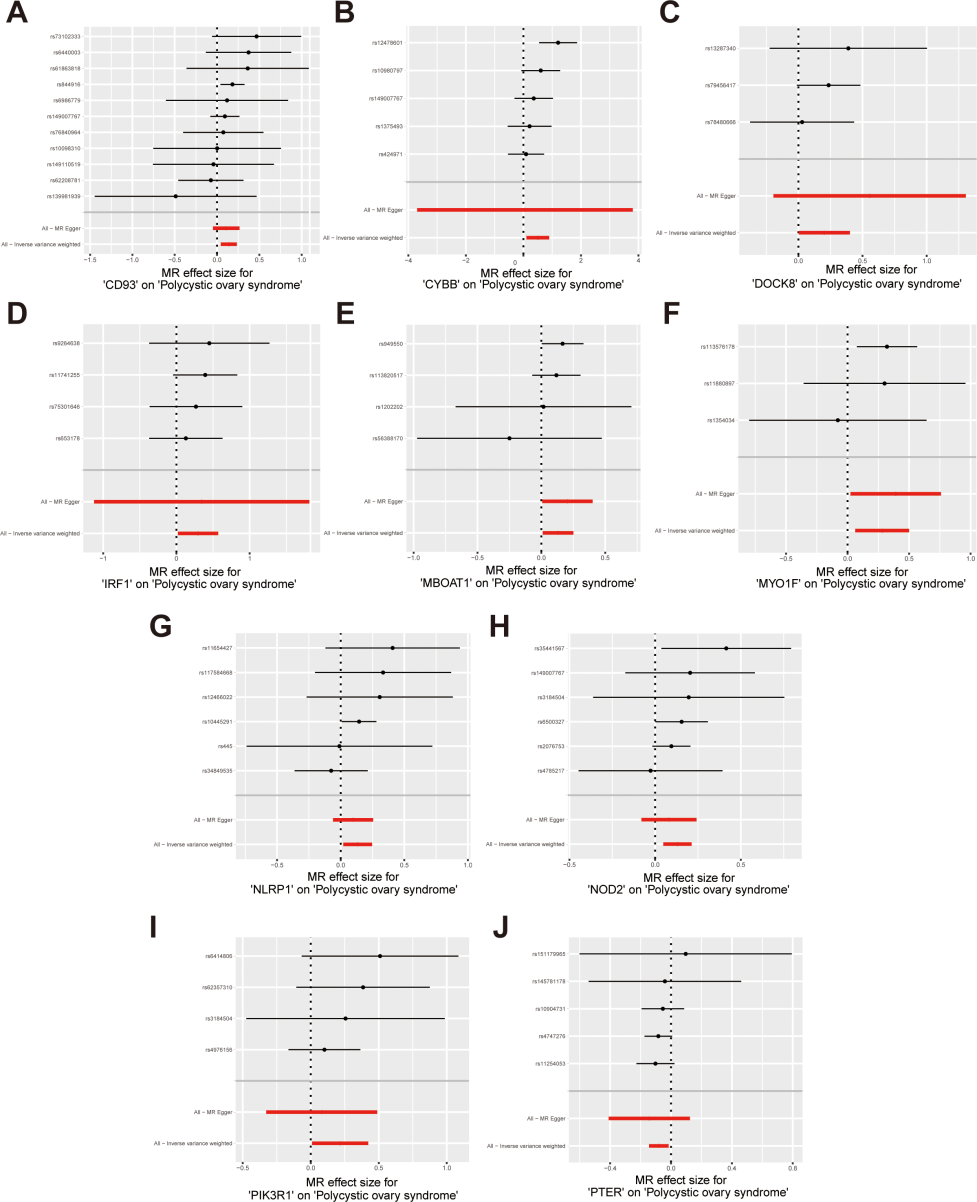
Forest plots illustrate the causal effects of hub genes on PCOS. **(A-I)** Forest plot of MR analysis results for CD93, CYBB, DOCK8, IRF1, MBOAT1, MYO1F, NLRP1, NOD2, PIK3R*1*; **(J)** Forest plot of MR analysis results for PTER on PCOS.

According to the hub gene screening criteria, 10 hub genes were obtained. Venn diagram showed 9 up-regulated hub genes CD93, CYBB, DOCK8, IRF1, MBOAT1, MYO1F, NLRP1, NOD2, PIK3R1, and 1 down-regulated hub gene PTER (**Figures 3A, B**). In the MR analysis, we identified potential causal relationships between 10 hub genes and PCOS. The results obtained from the IVW method in the MR analysis are presented below: CD93 [*P*= 0.004; *OR* 95%*CI*= 1.150 (1.046, 1.264)], CYBB [*P*= 0.013; *OR* 95%*CI*= 1.650 (1.113,2.447)], DOCK8 [*P*= 0.048; *OR* 95%*CI*= 1.223 (1.002,1.494)], IRF1 [*P*= 0.036; *OR* 95%*CI*= 1.343 (1.020,1.769)], MBOAT1 [*P*= 0.033; *OR* 95%*CI*= 1.140 (1.011,1.285)], MYO1F [*P*= 0.012; *OR* 95%*CI*= 1.325 (1.065,1.649)], NLRP1 [*P*= 0.020; *OR* 95%*CI*= 1.143 (1.021,1.280)], NOD2 [*P*= 0.002; *OR* 95%*CI*= 1.139 (1.049,1.237)], PIK3R1 [*P*= 0.040; OR 95%*CI*= 1.241 (1.010,1.526)], PTER [*P*= 0.015; *OR* 95%*CI*= 0.923 (0.866,0.984)] (**Figure 3C**). Additional MR methods are described in detail in the **Supplementary Table S11**. While the 95% *CIs* for the remaining four methods were wide, their estimated values were largely consistent with the direction indicated by the IVW method (**Figures 4**, **5**).

**Figure 6:**
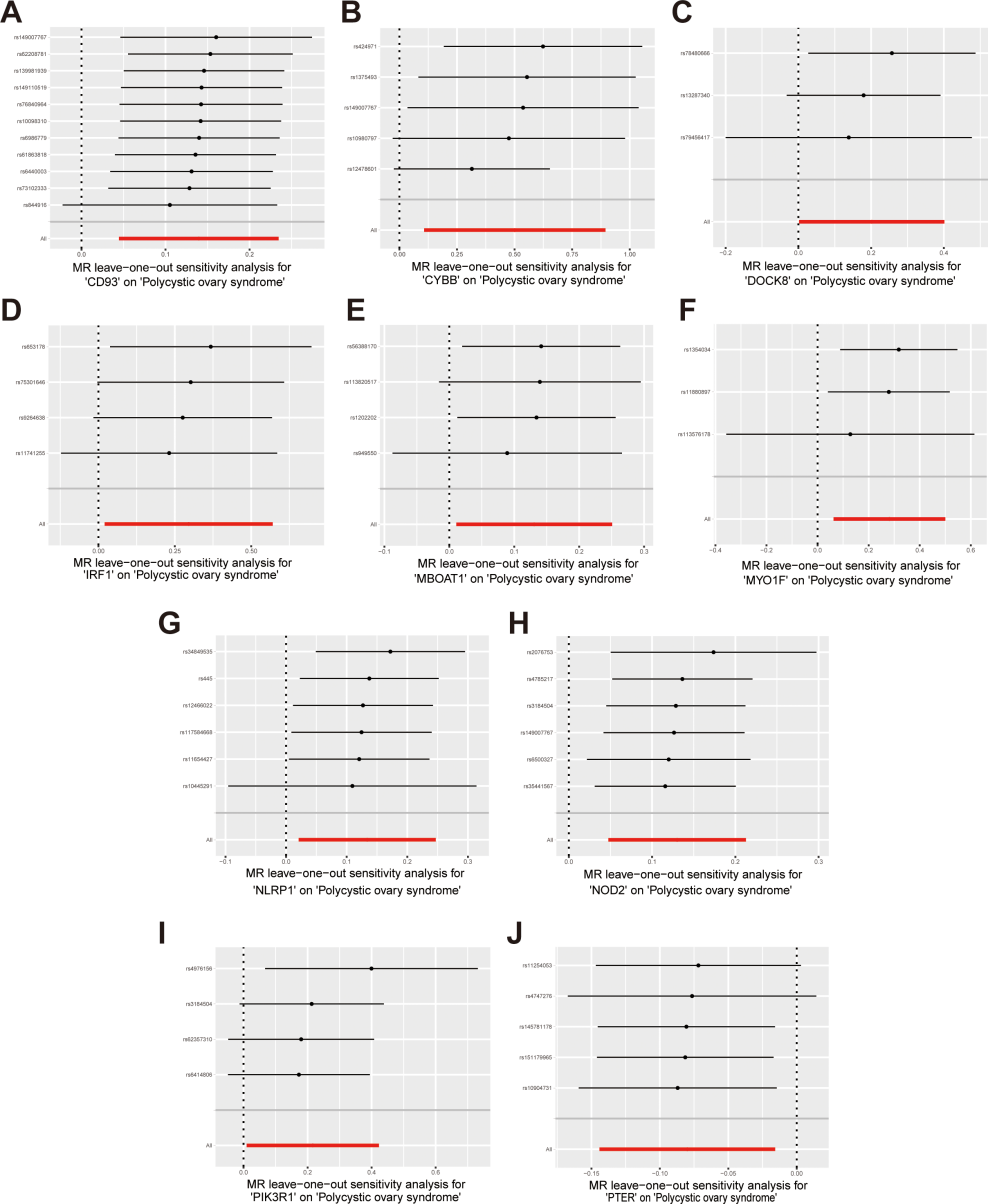
Forest plots of leave-one-out sensitivity analysis provide evidence supporting the validity of the IVW results. **(A)** CD93 on PCOS; **(B)** CYBB on PCOS; **(C)** DOCK8 on PCOS; **(D)** IRF1 on PCOS; **(E)** MBOAT1 on PCOS; **(F)** MYO1F on PCOS; **(G)** NLRP1 on PCOS; **(H)** NOD2 on PCOS; **(I)** PIK3R1 on PCOS; **(J)** PTER on PCOS.

Sensitivity analysis was performed to evaluate heterogeneity and pleiotropy using the Cochran Q method and MR-Egger. The findings of these analyses are presented in **Supplementary Table S12**, indicated that all p-values were above 0.05, providing no evidence of heterogeneity or pleiotropy in the SNPs examined. To further evaluate the stability of our results, we conducted a leave-one-out analysis, which demonstrated consistent findings (**Figure 6**). Moreover, the funnel plot displayed no indications of horizontal pleiotropy in our study (**Supplementary Figure S1**), further supporting the reliability of our results.

### Bioinformatics analyses of hub genes

**Figure 7:**
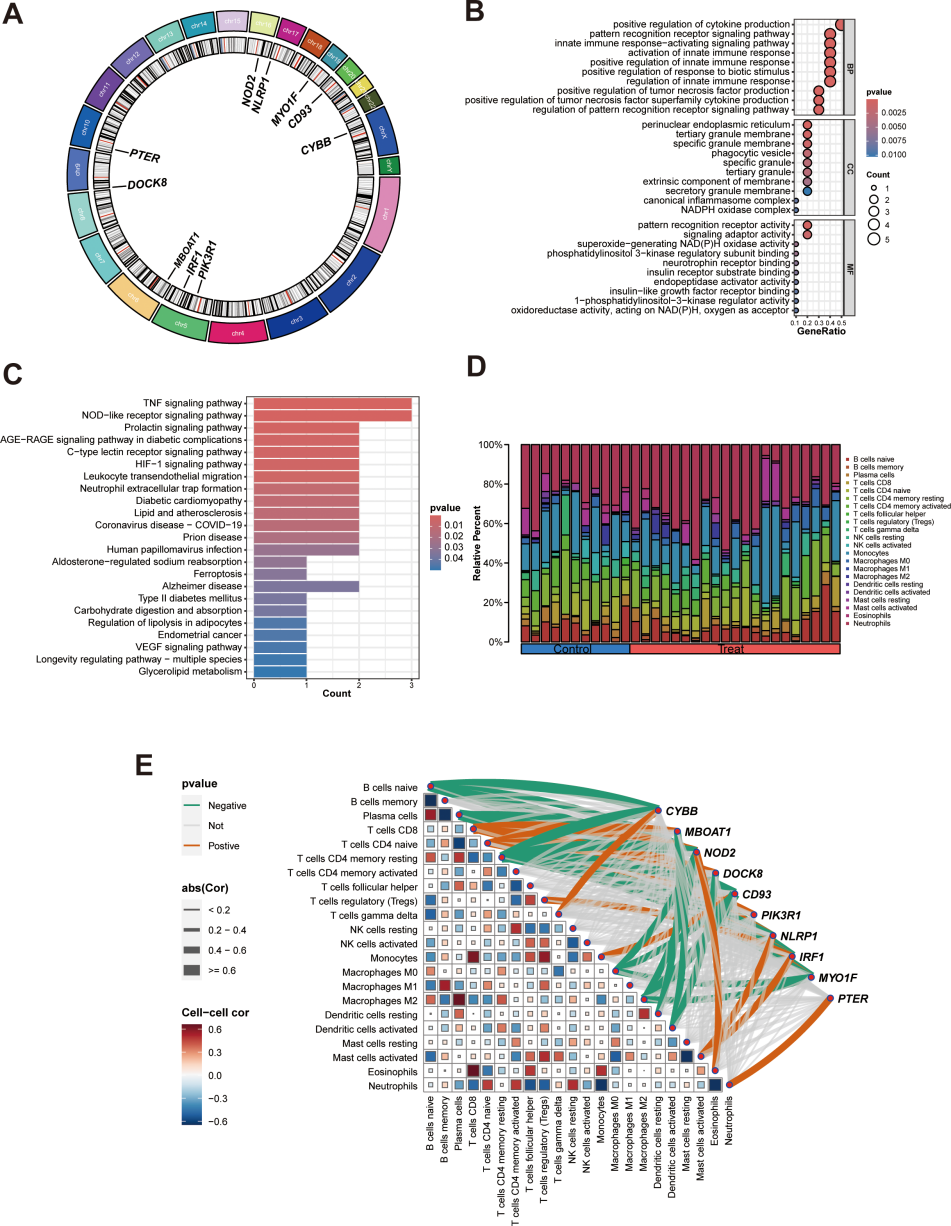
Bioinformatics analyses of hub genes. **(A)** Circos plot of hub genes; **(B)** GO enrichment analysis of hub genes; **(C)** KEGG enrichment analysis of the hub genes; **(D)** A Composition of infiltration immune cells in the PCOS and control groups; **(E)**. Heat map of correlation between hub genes and immune cells, green and red lines indicated positive and negative correlations, respectively, with thicker lines indicating stronger correlations.

Circos plot showed the distribution of hub genes in chromosomes (**Figure 7A**). To gain insights into the potential biological roles of these genes, enrichment analysis was performed. GO analysis showed that hub genes were mainly enriched in positive regulation of cytokine production, pattern recognition receptor signaling pathway, innate immune response-activating signaling pathway, activation of innate immune response, positive regulation of tumor necrosis factor production, positive regulation of innate immune response, perinuclear endoplasmic reticulum, pattern recognition receptor activity (**Figure 7B**). KEGG analysis revealed that hub genes were predominantly enriched in pathways including TNF signaling pathway, NOD-like receptor signaling pathway, Prolactin signaling pathway, AGE-RAGE signaling pathway in diabetic complications, C-type lectin receptor signaling pathway, HIF-1 signaling pathway (**Figure 7C**). These enrichment results suggested hub genes may be closely related to immune inflammation in the occurrence and progression of PCOS, so we further conducted immune infiltration analysis.

Immune function analysis showed that changes of hub genes expression greatly affected immune cells and immune function scores, suggesting that hub genes may affect the occurrence of PCOS by affecting immune function (**Supplementary Figure S2**). Then the infiltration immune cells around GCs was predicted using the CIBERSORT algorithm, as illustrated in **Figure 7D**. Compared with other immune cells, monocytes and neutrophils were found to be the predominant immune cells in both PCOS and control groups. Correlation analysis was conducted to evaluate the relationships of hub genes with infiltration immune cells (**Figure 7E**, **Supplementary Table S13**). We identified that each hub genes had its own associated (positive or negative) immune infiltrating cell.

### Validation of hub gene expression in PCOS rat models

**Figure 8:**
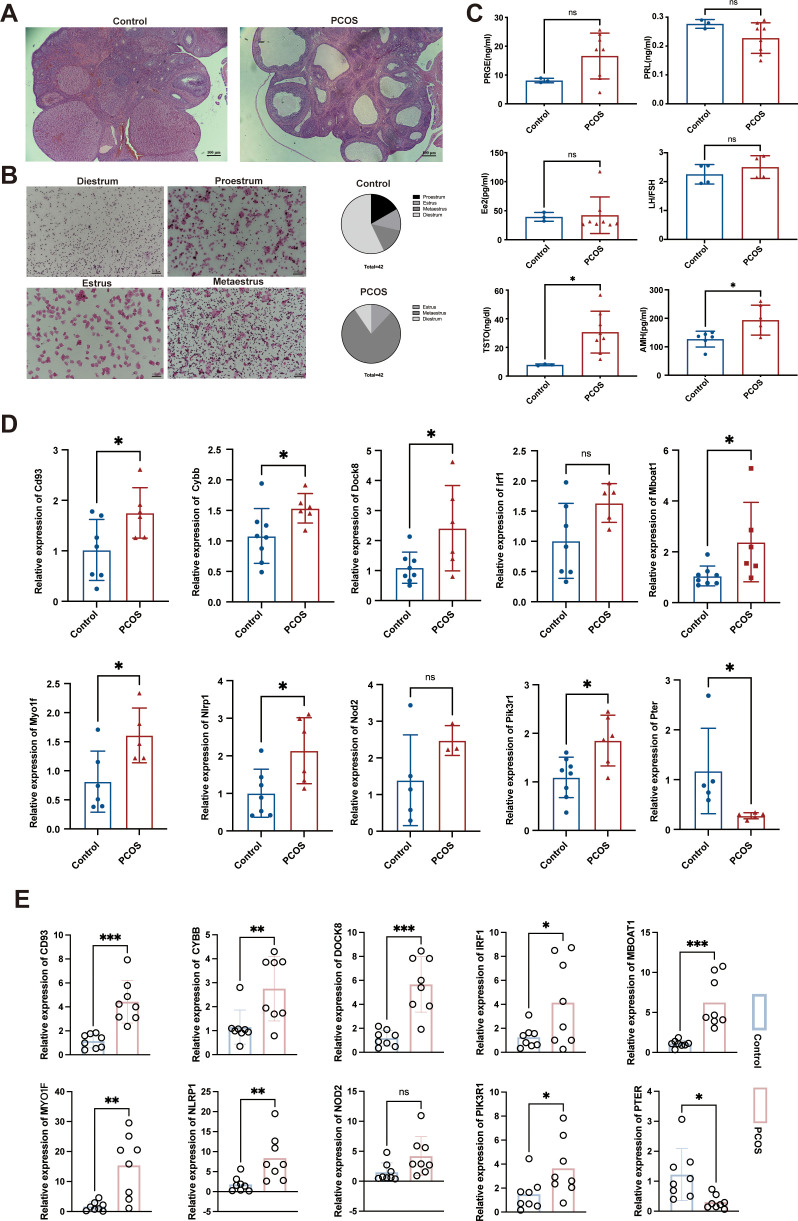
Construction of the DHEA- induced PCOS rat model and measurement of hub gene expression. **(A)** HE staining results of the ovarian tissues in various groups of rats, magnification: 40×; **(B)** Estrus status of rats in the DHEA and control groups; **(C)** Levels of sex hormones in rat serum; **(D)** Expression levels of Cd93, Cybb, Dock8, Irf1, Mboat1, Myo1f, Nlrp1, Nod2, Pik3r1, and Pter in rat ovarian tissue, as determined by qRT-PCR; **(E)** Expression levels of CD93, CYBB, DOCK8, IRF1, MBOAT1, MYO1F, NLRP1, NOD2, PIK3R1, and PTER in clinical serum samples as determined by qRT-PCR. PRGE, progesterone; PRL, prolactin; E2, estradiol; FSH, follicle-stimulating hormone; LH, luteinizing hormone; T, testosterone; AMH, anti-Mullerian hormone. The adjusted p value is ns, which is not significant. *P < 0.05, **P < 0.01 and ***P < 0.001.

Histological observation showed that the ovaries of DHEA-induced rat models exhibited multiple cystic follicles, reduced corpora lutea, and significant disruption of estrous cycle, primarily characterized by prolonged estrus (**Figures 8A, B**). Then, we also detected the levels of sex hormones (PRGE, PRL, E2, FSH, LH, T and AMH) in rat serum. Hormone testing results indicated significantly elevated levels of testosterone and AMH in PCOS rats (*P*<0.05) (**Figure 8C**). Therefore, subcutaneous injection of DHEA for 23 days can establish a PCOS model in rats.

Compared with the control group rats, the Cd93, Cybb, Dock8, Mboat1, Myo1f, Nlrp1, Pik3r1 mRNA levels were significantly elevated and the Pter mRNA level was significantly reduced in the PCOS group rats (*P* < 0.05). Although not reaching statistical significance for Irf1 and Nod2 (*P* > 0.05), we noted that they were elevated in the PCOS group (**Figure 8D**).

In clinical serum samples, CD93, DOCK8, MBOAT1 (*P* < 0.001), CYBB, MYO1F, NLRP1(*P* < 0.01), IRF1, and PIK3R1 (*P* < 0.05) mRNA levels were significantly elevated and the PTER mRNA level was significantly reduced in the PCOS patients (*P* < 0.05). Additionally, NOD2 mRNA level showed an increase in PCOS patients without statistical significance (*P* > 0.05) (**Figure 8E**). Overall, these results validate the predictions obtained from the combination analysis of bioinformatics and MR, validating these genes’ association with PCOS in both rat models and human patients.

## Discussion

Our study integrated individual-level and aggregated datasets from GEO as well as GWAS datasets, employing comprehensive bioinformatics analysis and MR methods to jointly screen hub genes for PCOS. We demonstrated the roles of hub genes in PCOS, analyzed their causal relationships with PCOS from a genetic perspective, and constructed animal models to further verify their expression in both animal models and clinical serum samples. By employing comprehensive genetic approaches with the aid of extensive GWAS summary data, our analysis presents suggestive evidence of the potential contribution of hub genes on PCOS risk. To our knowledge, this is the first MR analysis investigating the causal relationships between hub genes and PCOS. Previous studies mainly screened differential genes using bioinformatics methods alone, and the approach of combining MR to select hub genes has not been reported in this disease. Through the utilization of SNPs as IVs and the integration of various MR methods, we found that the expression of CD93, CYBB, DOCK8, IRF1, MBOAT1, MYO1F, NLRP1, NOD2, PIK3R1 were associated with an increased risk of PCOS, while the expression of PTER was associated with a decreased risk of PCOS.

CD93 is a phagocytic receptor, studies have indicated a close association between the occurrence of PCOS and cellular apoptosis (28, 29). Yefimova et al. found that granulosa cells can influence oocyte apoptosis through unconventional autophagy-assisted phagocytosis (30). We speculated CD93’s association as a risk factor in PCOS may be related to its involvement in promoting phagocytic activity. Nevertheless, additional research is warranted to validate this hypothesis. CYBB, also known as NOX2, has been studied in PCOS. Zhou et al. found that metformin treatment in PCOS may decrease NOX2 levels by upregulating miR-670-3p, thereby reducing ROS production, inhibiting NLRP3 inflammasome activation, and improving KGN cell pyroptosis (31). Additionally, Shen et al. discovered elevated levels of the purinergic receptor P2X7 in PCOS samples, which activated NOX2 and contributed to granulosa cell inflammation and apoptosis (32). Both studies demonstrated a higher trend of NOX2 levels in PCOS, which is consistent with our research findings. IRF1 functions as a transcription factor that exerts a positive regulatory effect on type I interferon. Previous studies have revealed that IRF1 may contribute to the risk of PCOS through developmental mechanisms (33), and it exhibits high expression levels in ovarian cancer (34). Liu et al. speculated that abnormal expression of IRF1 during late-stage fetal ovarian development may be associated with the fetal origins of PCOS. The PIK3R1 gene encodes the 85-kDa regulatory subunit of phosphoinositide 3-kinase. Our results indicated that PIK3R1 is a risk factor for PCOS. Previous studies have suggested that this gene is considered a potential pathogenic variant in PCOS and is associated with severe insulin resistance, which aligns with the trends observed in our findings (35, 36).

Our study identified a correlation between heightened levels of DOCK8 and an increased risk of PCOS. DOCK8 belongs to the DOCK protein family and is highly expressed in B cells and T cells, making it widely used in immune system research (37). While no previous studies have directly linked DOCK8 to PCOS, our analysis found that DOCK8 showed a negative correlation with dendritic cells activated (R^2^=-0.51, *P*<0.05) and a positive correlation with T cells CD4 naive (R^2^ = 0.46, *P*<0.05). A recent large-scale MR analysis investigating the relationship between immune cells and PCOS also suggested the involvement of various immune cells in PCOS pathogenesis (38). Furthermore, our findings were validated through an animal model and clinical samples, demonstrating its upregulation in the disease context. MBOAT1 is a lipid metabolism-related gene, previous study has reported the significant role of lipid metabolism in oocyte development (39). We hypothesized that MBOAT1 may contribute to potential metabolic imbalances in PCOS, thereby affecting the normal development of oocytes and subsequently impacting the reproductive capacity in PCOS. PCOS is commonly associated with an inflammatory state (40). MYO1F was thought to enhance cell adhesion and migration, which are crucial steps in the recruitment of neutrophils to inflammatory sites during the inflammatory process, it played a significant role in this process (41, 42). NLRP1 is an inflammasome complex that, when aberrantly activated, can lead to chronic inflammation, and its mutations have been implicated in inflammatory diseases in both mice and humans (43). Additionally, NOD2 is believed to induce pro-inflammatory responses (44). We found that these three pathogenic genes are associated with promoting inflammatory reactions. In the KEGG enrichment analysis, TNF signaling pathway and NOD-like receptor signaling pathway ranked among the top 2. Although there have been no reports directly elucidating the relationship between MYO1F, NLRP1, NOD2 and PCOS, we believe that our results still have a theoretical basis.

PTER was the only PCOS-protective gene we identified through our screening process. Currently, there is very limited research available on its functionality, and specifically, studies related to its association with the ovary are lacking. Our animal model and clinical serum sample validation confirmed a downward trend in PTER expression. However, our immune infiltration analysis indicated a positive correlation between PTER and neutrophils (R^2^ = 0.46, P<0.05). We acknowledge that neutrophils typically participate in acute inflammatory responses, while PCOS is more commonly associated with a chronic inflammatory state (45). Therefore, our results are not contradictory; however, further research is still required to establish additional theoretical basis for this conclusion.

This study possesses several strengths, including the comprehensive exploration of the relationship between hub genes and PCOS from various perspectives through the utilization of multiple methods such as bioinformatics analysis and MR methods. These approaches ensure a comprehensive and dependable research outcome. Furthermore, this study relied on publicly accessible databases and a substantial volume of gene expression data, thus establishing a strong basis for the research. Lastly, various MR methods were employed to ensure the credibility and precision of the research findings. However, it is important to acknowledge the limitations of this study. First and foremost, although MR analysis provides insights into causal relationships between exposures and outcomes, it cannot substitute objective clinical trials. Therefore, additional research is needed to validate the potential associations between hub genes and PCOS risk. Although initial validation was conducted using both clinical samples and an animal model, further in-depth experiments conducted *in vitro* and *in vivo* remain necessary to deepen our understanding. We recognize that in silico analyses play a critical role in identifying potential targets and generating initial hypotheses; however, they have inherent limitations, particularly without direct experimental validation. We are committed to ongoing experimental validation in future studies to strengthen the clinical applicability of our findings. Furthermore, while the GEO database includes populations from Asia and North America, the GWAS database used data predominantly from European populations. Considering the genetic heterogeneity observed among diverse ethnic groups, it is important to acknowledge that the findings of this study may vary across different populations. Future research should include subgroup analyses of different populations and further explore the genetic characteristics of various PCOS subtypes (such as lean vs. obese, BMI, and endocrine profile alterations) to better understand the heterogeneity of PCOS and to derive more comprehensive and representative conclusions. Additionally, we recognize that specific epigenetic markers associated with PCOS complications, such as hypertension, diabetes, and endometrial cancer, may be crucial for prognosis and identifying complications. Therefore, future studies will incorporate these specific complications to enhance the clinical applicability of our findings. MR can only provide insights into causal relationships from a genetic perspective and cannot establish causal relationships from environmental factors.

In summary, our bioinformatics combined with MR analysis identified CD93, CYBB, DOCK8, IRF1, MBOAT1, MYO1F, NLRP1, NOD2, PIK3R1 as risk factors for PCOS, while PTER was found to be protective. This finding contributes to clinical decision-making in disease diagnosis, prognosis, and treatment strategies, offering new avenues for drug development. However, the development of PCOS involves multiple complex factors and mechanisms, and the intricate interactions between hub genes and PCOS at the epigenetic level require further investigation.

## Data Availability

The datasets presented in this study can be found in online repositories. The names of the repository/repositories and accession number(s) can be found in the article/**Supplementary Material**. The acquisition of GEO database was obtained from the publicly accessible online platform (https://www.ncbi.nlm.nih.gov/geo), and the GWAS summary data utilized in this study were obtained from a publicly accessible online platform (https://gwas.mrcieu.ac.uk/).

## Supplementary material

Supplementary Figure S1 Funnel plots demonstrate the absence of pleiotropy among the included SNPs. **(A)** CD93 on PCOS; **(B)** CYBB on PCOS; **(C)** DOCK8 on PCOS; **(D)** IRF1 on PCOS; **(E)** MBOAT1 on PCOS; **(F)** MYO1F on PCOS; **(G)** NLRP1 on PCOS; **(H)** NOD2 on PCOS; **(I)** PIK3R1 on PCOS; **(J)** PTER on PCOS.

Supplementary Figure S2 The relationship between the hub genes expression and immune infiltrating cells and immune-related functions. **(A-J)** The relationship between CD93, CYBB, DOCK8, IRF1, MBOAT1, MYO1F, NLRP1, NOD2, PIK3R1, PTER expression and immune cells and immune-related functions. The adjusted p value is ns, which is not significant. *P < 0.05; ** P < 0.01; *** P < 0.001.
