# Supplementary figures and images for: Unveiling the molecular landscape of PCOS: identifying hub genes and causal relationships through bioinformatics and Mendelian randomization
Source: Front Endocrinol (Lausanne). 2024 Dec 13;15:1431200. doi: 10.3389/fendo.2024.1431200 (PMC11671271; doi:10.3389/fendo.2024.1431200)

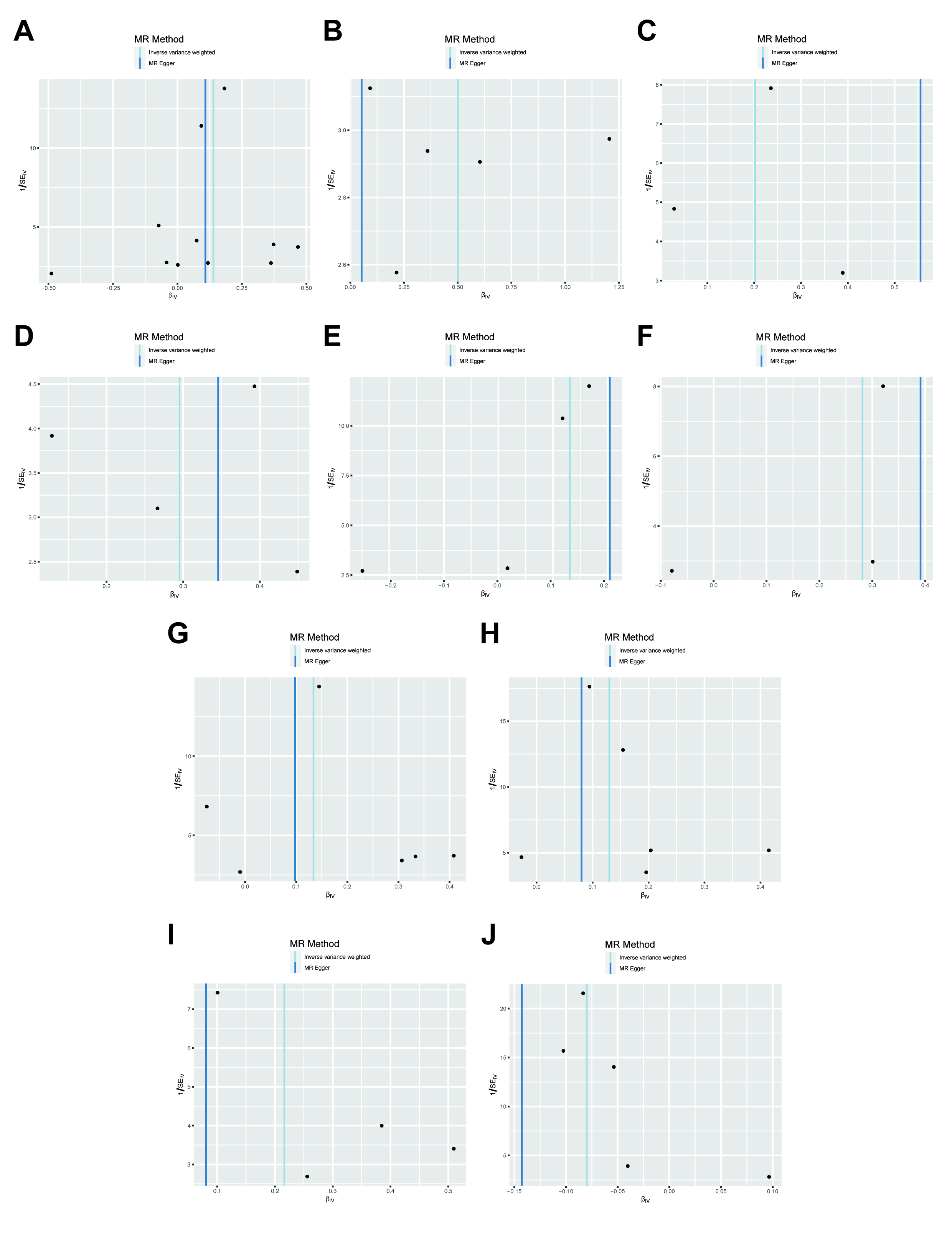

Supplement: Supplementary Figure S1 — Funnel plots demonstrate the absence of pleiotropy among the included SNPs. (A) CD93 on PCOS; (B) CYBB on PCOS; (C) DOCK8 on PCOS; (D) IRF1 on PCOS; (E) MBOAT1 on PCOS; (F) MYO1F on PCOS; (G) NLRP1 on PCOS; (H) NOD2 on PCOS; (I) PIK3R1 on PCOS; (J) PTER on PCOS. [file Image1.tif]

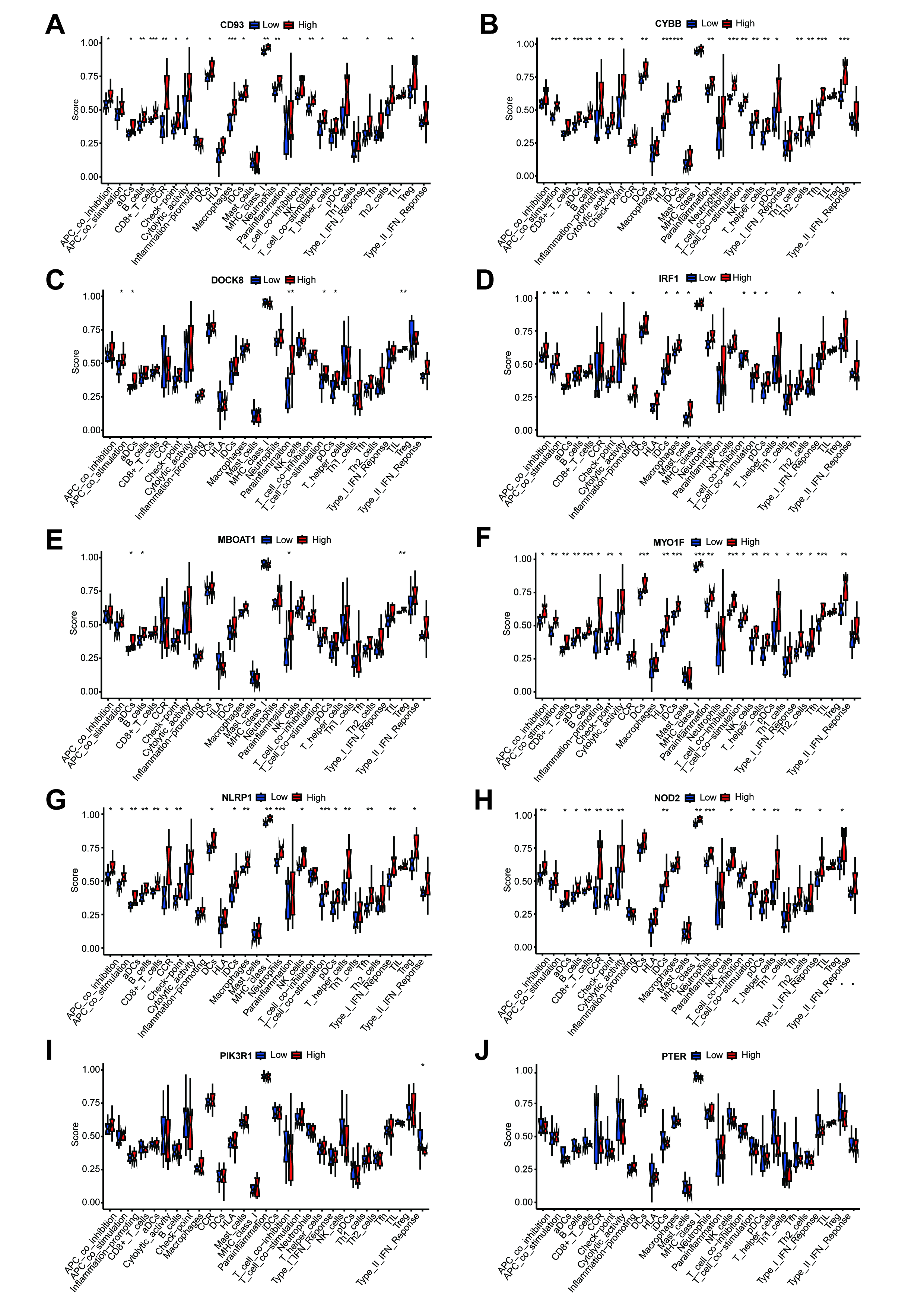

Supplement: Supplementary Figure S2 — The relationship between the hub genes expression and immune infiltrating cells and immune-related functions. (A-J) The relationship between CD93, CYBB, DOCK8, IRF1, MBOAT1, MYO1F, NLRP1, NOD2, PIK3R1, PTER expression and immune cells and immune-related functions. The adjusted p value is ns, which is not significant. *P < 0.05; ** P < 0.01; *** P < 0.001. [file Image2.tif]
